# ^1^H-NMR-Based Metabonomics of the Protective Effect of *Coptis chinensis* and Berberine on Cinnabar-Induced Hepatotoxicity and Nephrotoxicity in Rats

**DOI:** 10.3390/molecules22111855

**Published:** 2017-11-02

**Authors:** Guangyue Su, Haifeng Wang, Yuxian Gao, Gang Chen, Yuehu Pei, Jiao Bai

**Affiliations:** 1 Department of Traditional Chinese Materia Medica, Shenyang Pharmaceutical University, Shenyang 110016, China; wanghaifeng0310@163.com (H.W.); akid007@icloud.com (Y.G.); chengang1152001@163.com (G.C.); peiyueh@vip.163.com (Y.P.); 2 Key Laboratory of Structure-Based Drug Design & Discovery, Ministry of Education, Shenyang Pharmaceutical University, Shenyang 110016, China; 3 School of Functional Food and Wine, Shenyang Pharmaceutical University, Shenyang 110016, China; suggyy@163.com

**Keywords:** metabonomics, NMR, detoxification, *Coptis chinensis*, berberine, cinnabar

## Abstract

*Coptis chinensis* Franch has been used in Traditional Chinese Medicine (TCM) for treating infectious and inflammatory diseases for over two thousand years. Berberine (BN), an isoquinoline alkaloid, is the main component of *Coptis chinensis*. The pharmacological basis for its therapeutic effects, which include hepatoprotective effects on liver injuries, has been studied intensively, yet the therapy of liver injuries and underlying mechanism remain unclear. We investigated the detoxification mechanism of *Coptis chinensis* and berberine using metabolomics of urine and serum in the present study. After the treatment with *Coptis chinensis* and berberine, compared with the cinnabar group, *Coptis chinensis* and berberine can regulate the concentration of the endogenous metabolites. PLS-DA score plots demonstrated that the urine and serum metabolic profiles in rats of the *Coptis chinensis* and berberine groups were similar those of the control group, yet remarkably apart from the cinnabar group. The mechanism may be related to the endogenous metabolites including energy metabolism, amino acid metabolism and metabolism of intestinal flora in rats. Meanwhile, liver and kidney histopathology examinations and serum clinical chemistry analysis verified the experimental results of metabonomics.

## 1. Introduction

Metals (minerals) have been used in Traditional Chinese Medicine for a long time. According to the Pharmacopoeia of China (2015), ten kinds of minerals are listed, including cinnabar (HgS) and realgar (As_4_S_4_). Cinnabar (96% as HgS) has long been used for its sedative and hypnotic effects in TCM prescriptions [1,2], such as Zhusha Anshen Wan (ZSASW) [3], An-Gong-Niu-Huang Wan [4], etc. Although cinnabar has many special medical effects, the safety of cinnabar is becoming of more and more concern to the public due to the toxic effects caused by the high mercury content [5]. The actual Hg content in ZSASW is about 11–13%, i.e., 110 mg Hg/g, which is 110,000 times higher than the European Drug and Food Standards (1 μg Hg/g) [6]. Consumption of food and drugs can cause cinnabar and other minerals to enter the body, and in some cases can cause liver and kidney injuries [3]. Therefore, it is of great significance to find a drug or effective component which has a protective effect on liver and kidney toxicity induced by cinnabar.

*Coptidis chinensis* (CR, *Huanglian* in Chinese) is a Chinese herbal medicine used in Traditional Chinese Medicine (TCM) for thousands years to treat syndromes for its detoxifying and heat-clearing effects [7,8]. For instance, *Huanglian Jiedu* decoction has been used for hepatitis and liver dysfunction therapy [9]. It was reported that *Coptis chinensis* had hepatoprotective effects on carbon tetrachloride-induced liver injury through regulating the abnormal metabolism of transaminase [10], yet the mechanism of its liver protective effects remains unexplored. Many studies have shown that many biological activities of *Coptis chinensis* are due to its main chemical component berberine [11,12,13]. Berberine (molecular formula C_20_H_19_NO_5_), an isoquinoline alkaloid, is isolated from *Coptidis* rhizome [14]. Berberine has many pharmacological and biological activities and previous studies have shown that it has beneficial effects such as hypoglycemic, antioxidant and anti-inflammatory activity [15]. Recent studies have found that berberine has a good protective effect on liver and kidney injury caused by mercury by increasing the expression of Bcl-2 protein in liver and kidney [16]. However, no study of the protective effect of *Coptis chinensis* and berberine on the metabolic pathways of liver and kidney has been reported.

Metabonomics is the science of comprehensively profiling small molecule metabolites in cells, tissues, or whole organisms, the application of which has led to an understanding of the pathophysiologic mechanisms of cardiometabolic diseases, defining predictive biomarkers for those diseases, and also assessing the efficacious effects of administered drugs. As an important part of systems biology, metabonomics refers to a qualitative and quantitative analysis of all low molecular-weight metabolites produced by cells, tissues or organisms within a specific period [17]. It has been well known that a disease can cause pathological and physiological changes in the body, which may cause subsequent changes in these low-molecular-weight metabolites [18]. Metabonomics these are not interchangeable- do not mix has become more and more useful for the characterization of the metabolic changes and the biomarkers involved in toxicological mechanisms [19,20]. NMR spectroscopy, which requires minimal sample preparation [21], provides a rapid, non-destructive and high-throughput method, and is been one of the most powerful techniques used in metabonomics studies.

To elucidate the underlying mechanisms of the protective effects of *Coptis chinensis* and berberine on liver and kidney injury caused by cinnabar, in the present study a ^1^H-NMR-based metabolomic method was applied to evaluate the systemic metabolic consequences. 

## 2. Results

### 2.1. Biochemical Characteristics and Histopathology

The clinical biochemical results showed the parameters in the serum samples from the rats treated with *Coptis chinensis* + cinnabar and berberine + cinnabar were different from those of the cinnabar group on day 10 of continuous administration (Table 1).

The elevated level of alanine aminotransferase (ALT) and aminotransferase (AST) in the cinnabar-treated groups indicated that the liver was damaged, releasing ALT and AST into the blood [22]. Furthermore, the creatinine (CREA) concentration was significantly increased, which reflected a renal function impairment. Compared to the cinnabar group, the *Coptis chinensis* + cinnabar and berberine + cinnabar groups showed milder and diminished toxic symptoms. The distributions of ALT values in the *Coptis chinensis* and berberine groups were less diffuse and closer to the mean value of the control group. The biochemical characteristics demonstrated that *Coptis chinensis* and berberine could thus effectively reduce the cinnabar-caused injury in rats.

**Figure 1:**
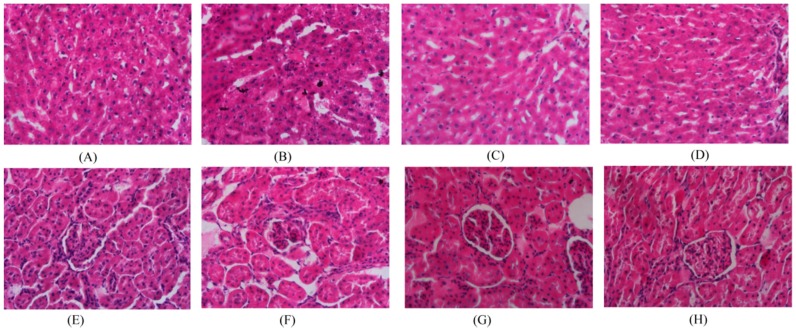
Kidney and liver histopathology of rats: Liver: control (**A**); cinnabar (**B**); *Coptis chinensis* group (**C**); berberine group (**D**); Kidney: control (**E**); cinnabar (**F**); *Coptis chinensis* group (**G**); berberine group (**H**).

Histopathological examination revealed that the main lesions showed diffuse hepatocyte degeneration, necrosis, apoptosis and significant swelling (ballooning degeneration) in the cinnabar group (Figure 1). By contrast, no liver and renal damage was observed in the *Coptis chinensis* and berberine groups.

### 2.2. Analysis of Urine Sample ^1^H-NMR Spectroscopic

**Figure 2:**
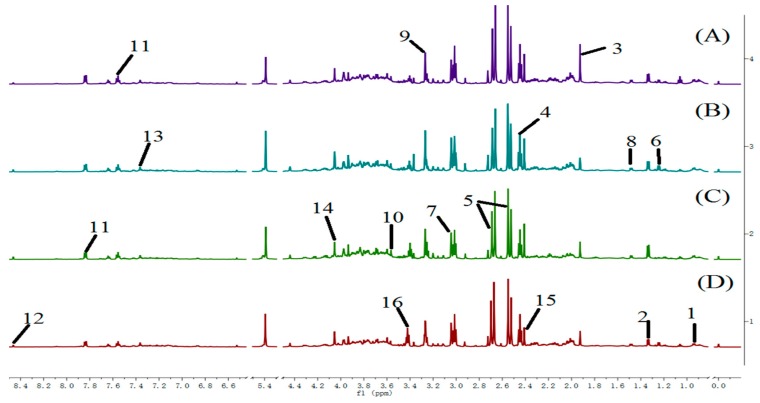
600 MHz ^1^H-NMR spectra of urine obtained from rats from the control (**A**); cinnabar (**B**); *Coptis chinensis* (**C**) and berberine groupc (**D**). Key: 1, leucine + isoleucine; 2, 3-hydroxybutyrate (3-HB); 3, acetate; 4, 2-ketoglutarate (2-OG); 5, citrate; 6, lactate; 7, creatine; 8, alanine; 9, trimethylamine-*N*-oxide (TMAO); 10, glycine; 11, hippurate; 12, formate; 13, phenylalanine; 14, creatinine; 15, succinate; 16, taurine.

Figure 2 shows ^1^H-NMR spectra of urine from rats in each group. Eighteen endogenous metabolites, i.e., lactate, valine, alanine, acetate, pyruvate, succinate, α-ketoglutarate, citrate, creatinine, choline, trimethylamine oxide (TMAO), betaine, taurine, glycine, fumarate, phenylalanine, hippurate and formate were identified in the samples.

**Figure 3:**
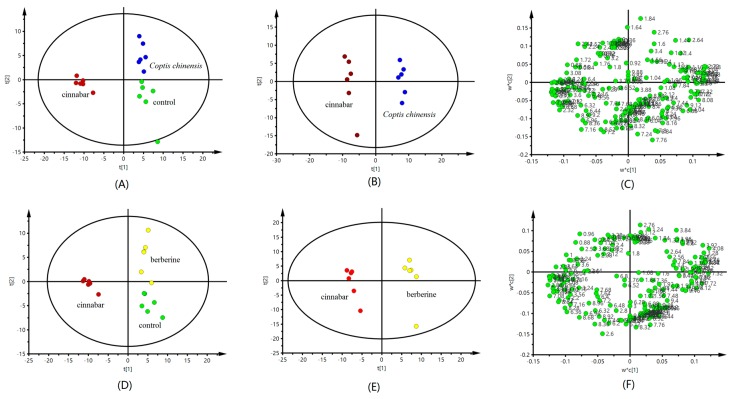
PLS-DA scores plot from control group, group cinnabar, group *Coptis chinensis* (**A**); group cinnabar and group *Coptis chinensis* (**B**); and corresponding loadings plot group cinnabar and group *Coptis chinensis* (**C**) derived from ^1^H-NMR spectra of urine. PLS-DA scores plot from control group, group cinnabar, group berberine (**D**); group cinnabar and group berberine (**E**); and corresponding loadings plot group cinnabar and group berberine (**F**) derived from ^1^H-NMR spectra. Key: control group (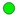); group Cinnabar (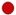); group *Coptis chinensis* (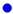); group berberine (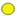).

PLS-DA analysis of the urine spectrometry results show that compared with the control and the cinnabar groups, both the *Coptis chinensis* and berberine ones appeared close to the control group, but significantly separated from the cinnabar group (Figure 3A,D). The PLS-DA scores plot also revealed that the *Coptis chinensis* and berberine groups could easily be distinguished from the cinnabar group along t1 (Figure 3B,E). The corresponding loadings plot (Figure 3C,F) combined with the VIP values from the pattern recognition model, screened out potential biomarkers for the differentiation of *Coptis chinensis*, berberine and cinnabar groups, The ^1^H-NMR-detected relative integral levels of metabolites indicating differences between the cinnabar group and *Coptis chinensis* and berberine groups are listed in Table 2.

Compared with cinnabar group, the level of α-oxoglutarate, citrate, TMAO and hippurate were significantly increased, and lactate, alanine, acetate, pyruvate, creatine, choline and taurine were significantly reduced in the *Coptis chinensis* and berberine groups.

### 2.3. ^1^H-NMR Spectroscopic Analysis of Serum Samples

**Figure 4:**
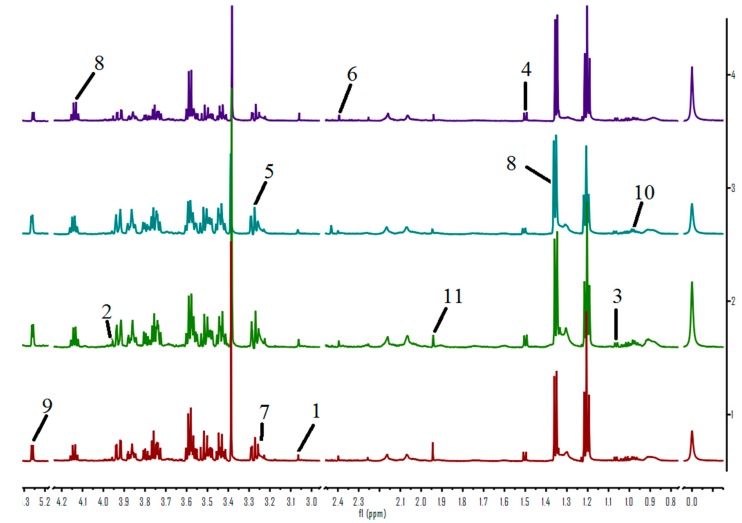
600 MHz spectra of serum obtained from rats from the control (**A**); cinnabar (**B**); *Coptis chinensis* group (**C**) and berberine groups (**D**). Key: 1, creatinine; 2, creatine; 3, valine; 4, alanine; 5, TMAO; 6, pyruvate; 7, choline; 8, lactate; 9, *α*-glucose; 10, leucine + isoleucine; 11, acetate.

Representative 600 MHz ^1^H-NMR spectra of serum obtained from controls and treated groups are shown in Figure 4. Endogenous metabolite assignments were based on chemical shifts reported in the literature [23].

**Figure 5:**
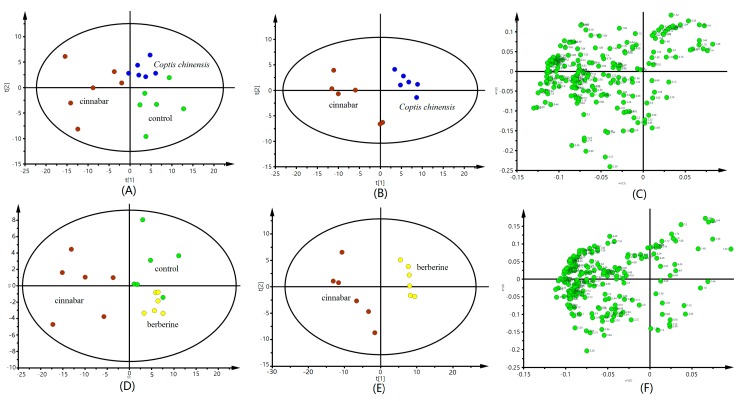
PLS-DA scores plot from control group, group cinnabar, group *Coptis chinensis* (**A**); group cinnabar and group *Coptis chinensis* (**B**); and corresponding loadings plot group cinnabar and group *Coptis chinensis* (**C**) derived from ^1^H-NMR spectra of serum. PLS-DA scores plot from control group, group cinnabar, group berberine (**D**); group cinnabar and group berberine (**E**); and corresponding loadings plot group cinnabar and group berberine (**F**) derived from ^1^H-NMR spectra. Key: control group (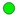); group Cinnabar (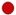); group *Coptis chinensis* (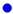); group berberine (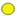).

The PLS-DA scores plots (Figure 5A,B,D,E) reveal an obvious separation of the control, *Coptis chinensis* and berberine groups from the cinnabar group. At the same time, we observed that there is no difference between the *Coptis chinensis*, berberine and control groups. The corresponding loadings plots, which show which metabolites contributed most to the separation of samples in the score plots, are presented in Figure 5C,F. The major biochemical changes identified in serum from the corresponding loadings plot and relative integral levels of metabolites (Table 3) showed the level of α-ketoglutaric acid, alanine, and TMAO were increased, and the levels of pyruvate, lactate, creatine, leucine, isoleucine and choline were decreased in the *Coptis chinensis* and berberine groups compared with the cinnabar group. These endogenous metabolites showed no significant differences in the *Coptis chinensis* and berberine groups compared with the control group.

### 2.4. Pathway Analysis

**Figure 6:**
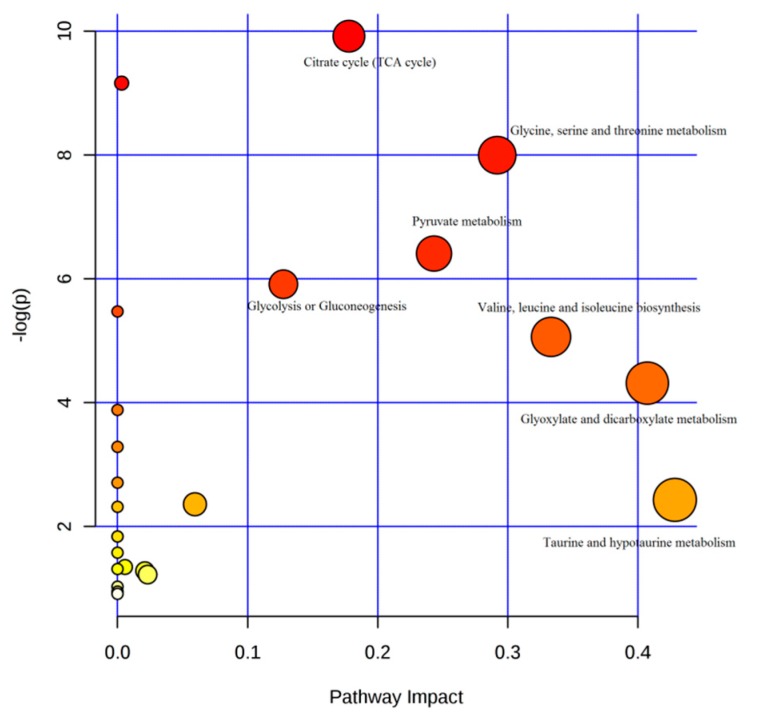
The pathway analysis summary.

In addition, to understand which pathways were the most relevant to the *Coptis chinensis* and berberine protective effect on acute hepatic and kidney injury, metabolic network was mapped by Metaboanalyst 3.0 (http://www.metaboanalyst.ca). A total of sixteen biomarkers were subjected to pathway analysis [23], and the associated metabolic pathways of each substance with their FDR values are summarized in Figure 6 and Table S1 (Supplementary Materials).

The pathways with the impact value > 0.1 were considered as the most relevant pathways for the liver injury. In this study, taurine and hypotaurine metabolism; glyoxylate and dicarboxylate metabolism; valine, leucine and isoleucine biosynthesis; glycine, serine and threonine metabolism; pyruvate metabolism; citrate cycle (TCA cycle) and glycolysis or gluconeogenesis pathways were important metabolic pathways with impact factors of 0.42, 0.40 0.33, 0.29, 0.24, 0.17, 0.012, respectively. The pathway analysis indicated that the alleviation effect of *Coptis chinensis* and berberine was connected to alterations in energy metabolism (TCA cycle), amino acid metabolism (valine, leucine and isoleucine biosynthesis).

## 3. Discussion

A large number of studies have reported that substances containing heavy metals such as HgS, cadmium (Cd), etc., can cause liver and kidney toxicity [24]. Heavy metals may enter the body through food, drugs, and decoration materials, etc. [1], and human intake of heavy metals for a long time will cause liver and kidney damage, and further lead to various types of liver diseases. In this work NMR metabonomics technologies were applied to elucidate the detoxification and mechanism of *Coptis chinensis* and its main component berberine on liver and kidney toxicity caused by cinnabar. The metabolites pathway analysis based on the potential biomarkers indicates that TCA cycle, lipid metabolism and amino acid metabolism were involved in the metabolic changes of urine and serum.

Lactate is a substance produced by the metabolism and movement of the human body, but its concentration generally does not rise. Only when the lactic acid production process increases, but cannot be excreted in time, will the concentration increase [25]. It is formed by the combination of pyruvate and hydrogen. If the energy metabolism of the rat body is normal, it does not produce any accumulation, breaks lactate down into water and carbon dioxide, and generates heat. The increase in the corresponding integral area shows that the metabolism of lactate in the cinnabar group has abnormally accelerated. In addition, the levels of acetate and creatine anhydride in serum of the cinnabar group were significantly increased. Increases in the amount of acetate in the serum indicate a fat metabolism disorder in the liver mitochondria [26]. Compared with the cinnabar group, the content of lactate, acetate and creatine in the urine of rats decreased to a level close to the control when the *Coptis chinensis* and berberine were administered. All the above results suggest that *Coptis chinensis* and berberine can improve the metabolism of hepatic mitochondria.

Trimethylamine oxide is the oxidation product of trimethylamine. It has the function of maintaining the concentration balance between the cell body fluid and the external body fluid environment. Studies have shown that the metabolism of trimethylamine oxide is related to the disorder of intestinal flora [27]. When the content of TMAO in the urine of rats was increased, it showed that the intestinal flora of rats was destroyed and the metabolism was disordered [28]. TMAO has been suggested as a link between gut microbiota and disease [29]. Gut microbiota play a critical role in several metabolic processes in the human body [30]. *Coptis chinensis* and berberine can significantly regulate the abnormal metabolism of trimethylamine oxide caused by cinnabar. The results suggest that *Coptis chinensis* and berberine ensure a good regulatory balance in the intestinal flora of the organism.

The main role of taurine is to participate in the regulation of environmental homeostasis in animals [31]. One of the standards for judging liver injury is the content of taurine in animal urine. Bile acid synthesis mainly contains taurine, and cholestasis in the liver can cause the bile acid concentration to increase, which can lead to an increase in the renal excretion of taurocholate acid. Taurine is likely to be a possible urinary marker of liver damage [32]. The level of taurine was restrained in the *Coptis chinensis* and berberine groups compared with the cinnabar group. The levels of taurine in the *Coptis chinensis* and berberine groups were close to those of the control group. This suggested that *Coptis chinensis* and berberine have protective effect on liver by regulating the taurine metabolism.

Creatine is synthesized in the liver and stored in the muscles. When the body’s energy is insufficient, creatine is phosphorylated and releases energy in skeletal muscle, and a series of changes are produced to produce creatinine. Creatinine is excreted through the glomerulus through the urine and is almost reabsorbed by the renal tubules. When the animal’s liver is damaged, it can lead to impaired energy metabolism and promote the production of creatinine. In addition, the increase of creatinine content is related to renal dysfunction [33]. A significant increase of creatine in serum was also observed after cinnabar adminstration. By contrast, after the administration of *Coptis chinensis* and berberine, the metabolism of creatine returned to normal, which implied that *Coptis chinensis* and berberine could prevent the liver and kidney damage induced by cinnabar.

Succinate, citrate and 2-OG are vital intermediates in the TCA cycle, which is the core metabolic pathway for energy, which promotes the oxidative decarboxylation of acetyl-CoA and produces reduced equivalents, FADH_2_ and NADH [34]. The level of the three endogenous metabolites were decreased in the cinnabar groups. However, succinate, citrate and 2-OG showed a reversion tendency in the *Coptis chinensis* and berberine groups, which indicated that the *Coptis chinensis* and berberine could restore the energy metabolism in rats.

## 4. Materials and Methods

### 4.1. Reagents and Drugs

Berberine chloride (purity of 99.0%), 2,2,3,3-deuterotrimethylsilyl propionic acid (TSP) and D_2_O were purchased from Norell Inc. (Vineland, NJ, USA). Phosphate buffer (pH 7.4) was prepared by mixing 0.2 M Na_2_HPO_4_ and 0.2 M NaH_2_PO_4_ (Sigma, St. Louis, MO, USA). Cinnabar and *Coptis chinensis*, were purchased from Liaoning Tongren Pharmaceutical Co, Ltd. (Shenyang, China) and authenticated by Professor Jincai Lu (School of Traditional Chinese Materia Medica, Shenyang Pharmaceutical University, China). *Coptis chinensis* was extracted by boiling distilled water for 2 h 3 times. After filtration, the aqueous solution of the extract was concentrated in a rotary evaporator and stored at 4 °C until use. To perform the experiments, *Coptis chinensis* was dissolved and vortexed for 2 min at room temperature.

### 4.2. Animals and Drug Administration

Adult male Wistar rats (SPF, 180 ± 20 g, animal license No. SCXK-(Military) 2013-004) were purchased from the Experimental Animal Center of Shenyang Pharmaceutical University. All the animal experiments were approved by the national legislations of China and local guidelines. After acclimatization to the environment for ten days, the rats were maintained under standard laboratory conditions (23 ± 1 °C, 45 ± 15% relative humidity, and 12 h/12 h light/dark cycle) in individual metabolic cages with freely available food and water. All the rats were randomly divided into four groups (*n* = 6): control group (injected water), cinnabar group (treated with cinnabar), *Coptis chinensis* group (treated with cinnabar and *Coptis chinensis* French), and berberine group (treated with cinnabar and berberine). All animals were treated by intragastric administration for consecutive eight days. The dosage of cinnabar was 1.8 g/kg according to the literature [3]. The dosage of *Coptis chinensis* was 2.7 g/kg [3], and the dosage of berberine waas 100 mg/kg according to the literature [16].

### 4.3. Sample Collection and Pretreatment

Urine samples were collected over dry ice in tubes overnight (from PM 7:00 to AM 7:00). And all the urine samples were immediately stored at −20 °C until NMR spectroscopic analysis was conducted. Blood samples from sacrificed rats were centrifuged at 14,000 rpm for 10 min at 4 °C. All the serums samples were stored at −80 °C for the NMR spectroscopic analysis and serum biochemistry assays.

### 4.4. Serum Biochemical Analysis and Histopathology Examination

The liver and kidney tissues were embedded in paraffin, and cut at 5 μm thickness. The sliced tissues were stained with hematoxylin and eosin (H&E), and examined by light microscopy (400×, Hitachi Medical Cor- poration, Tokyo, Japan). The serum biochemical examinations, including alanine aminotransferase (ALT), aspartate aminotransferase (AST), alkaline phosphatase (ALP), creatinine (CREA), triglyceride (TG), glucose (GLU) and total protein (TP), were performed using an automated clinical chemistry analyzer (AU5400; Beckman Coulter, USA). 

### 4.5. Preparation of Urine Samples and Serum Samples for ^1^H-NMR Spectroscopic Measurements

Urine samples (500 μL) were mixed with phosphate buffer (200 μL, 0.2 M, pH 7.4), and centrifuged at 15,000 rpm for 5 min to remove precipitated impurities. The supernatants (500 μL) were transferred into a 5 mm NMR tube containing TSP (100 μL, 0.1%, *w/v*,), and D_2_O (100 μL).

Serum samples (500 μL) were mixed with D_2_O (60 μL) and TSP (40 μL), and transferred to 5 mm NMR tubes. TSP acts as internal standard reference (*δ* 0.00 ppm) and D_2_O was used for locking the signal.

### 4.6. Data Reduction Analysis of ^1^H-NMR Spectra

Urine measurement conditions: ^1^H-NMR spectral measurements were acquired on an AV600 spectrometer (Bruker Biospin, Ettlingen, Germany) at 298 K. Typically, 32 free induction decays (FIDs) were collected into 64 k data points over a spectral width of 12,019.23 Hz with a relaxation delay of 3 s and an acquisition time of 2.73 s. Number of scans is 64.

Serum measurement conditions: ^1^H-NMR spectra of these samples were also recorded on the Bruker-AV600 spectrometer at 298 K. Pulse program is a 1D experiment with-T_2_ filter using the Carr-Purcell-Meiboom-Gill sequence. Thirty-two FIDs were collected into 64 k data points over a spectral width of 12,019.23 Hz with a relaxation delay of 3 s and an acquisition time of 2.73 s. Power level for presaturation is 50 dB, and number of scans is also 64.

All the serum and urine spectra were baseline corrected and manual phase adjustments of the spectrua were performed. Data sets were zero-filled to 64 k data points. Each urine or serum ^1^H-NMR spectrum was segmented into integrated regions at 0.04 ppm, corresponding to the chemical shifts *δ* 0.2–10.0 using MestReNova 11.0.2 (Mestrelab Research SL, Norwich, CT, USA). The *δ* 4.2–6.0 region was excluded to eliminate the effect of the water resonance and urea signals. The integral data were normalized to a constant unit area to reduce the effects of variation in concentration differences. Finally, the exported data was input into the SIMCA-P 13.0 software package (Umetrics AB, Umea, Sweden) for PLS-DA analysis. The goodness of fit for a model was evaluated according to the three quantitative parameters: R^2^X was the explained variation in X, R^2^Y was the explained variation in Y, and Q^2^Y was the predicted variation in Y. The range of the parameters was between 0 and 1, and the values approaching 1 indicate good fit for the model [35].

### 4.7. Statistical Analysis

All the data are expressed as mean ± standard deviation (SD). The significance testing was determined using one-way ANOVA followed by Dunnett’s test by SPSS 19.0 (IBM SPSS Inc, Chicago, IL, USA). *p* < 0.05 was considered as statistically significant.

## 5. Conclusions

The results clearly showed that the metabolic profiles of the *Coptis chinensis* and berberine groups were remarkably similar to those of the control group. In this work, the metabolite responses indicating the main liver and kidney protective effects of *Coptis chinensis* and berberine against liver and kidney damage induced by cinnabar were investigated by ^1^H-NMR-based metabonomics for the first time. ^1^H-NMR spectroscopy in conjunction with histopathology and clinical biochemical assays provided a special insight into the relationship between the metabolite changes and the toxicity to tissues. The corresponding biochemical pathways of energy metabolism, amino acid metabolism and gut microbiota disorder provided new suggestions for a systematic and holistic evaluation of the protective mechanism of Chinese herbal medicines on hepatic and renal injury caused by minerals.

## Figures and Tables

**Table 1:** Selected clinical biochemical parameters of serum.

Biochemical Parameters	Control	Group Cinnabar	Group of *Coptis chinensis*	Group of Berberine
AST (U/L)	97.40 ± 34.01	138.17 ± 25.69 *	88.80 ± 32.87 ^#^	90.00 ± 14.21 ^#^
ALT (U/L)	22.33 ± 3.39	26.00 ± 2.74	22.60 ± 5.13	20.17 ± 2.64
TP (g/L)	62.13 ± 5.33	63.12 ± 15.42	63.84 ± 7.12	56.60 ± 5.26
UREA (μmol/L)	8.60 ± 0.75	8.76 ± 2.07	9.62 ± 1.84	8.02 ± 1.58
CREA (μmol/L)	23.67 ± 11.55	38.25 ± 4.64 *	19.00 ± 2.55 ^#^	19.33 ± 3.44 ^#^
TG (mmol/L)	0.78 ± 0.17	0.86 ± 0.33	0.75 ± 0.27	0.57 ± 0.26
CHO (mmol/L)	1.32 ± 0.22	1.56 ± 0.32	1.63 ± 0.26	1.44 ± 0.18
GLU (mmol/L)	7.49 ± 1.77	10.38 ± 2.48 *	8.47 ± 1.80	10.49 ± 1.40 *

Data are presented as mean ± SD of six animals per group. * *p* < 0.05 when compared to control; ^#^ *p* < 0.05 when compared to cinnabar.

**Table 2:** ^1^H-NMR-relative integral levels of metabolites in urine samples of the control, cinnabar, *Coptis chinensis*, and berberine groups.

Metabolites	Chemical Shift (ppm)	Control Group	Cinnabar Group	*Coptis chinensis* Group	Berberine Group
Citrate	2.54 (d), 2.66 (d)	5.85 ± 0.42	4.28 ± 0.56 *	5.64 ± 0.62	5.93 ± 0.49 ^#^
Trimethylamine-*N*-oxide	3.27 (s)	2.84 ± 0.38	1.76 ± 0.45 *	2.88 ± 0.40	2.84 ± 0.44 ^#^
Succinate	2.41 (s)	2.57 ± 0.28	1.88 ± 0.22 *	2.36 ± 0.24	2.42 ± 0.19 ^#^
α-Oxoglutarate	2.47 (t), 3.01 (t)	2.24 ± 0.31	1.62 ± 0.25 *	2.22 ± 0.30 ^#^	2.16 ± 0.31
Hippurate	7.55 (t), 7.64 (t), 7.84 (d)	1.24 ± 0.13	0.68 ± 0.28 *	1.22 ± 0.12 ^#^	1.18 ± 0.15 ^#^
Lactate	1.32 (d), 4.14 (q)	1.05 ± 0.34	1.85 ± 0.27 *	1.07 ± 0.39	1.10 ± 0.32
Alanine	1.48 (d)	1.08 ± 0.22	1.13 ± 0.33	1.15 ± 0.12	1.03 ± 0.09
Acetate	1.93 (s)	1.64 ± 0.28	1.85 ± 0.31	1.59 ± 0.25	1.68 ± 0.16
Taurine	3.25 (t), 3.42 (t)	0.34 ± 0.08	0.66 ± 0.09 *	0.40 ± 0.05 ^#^	0.42 ± 0.08 ^#^
Creatineine	4.06 (s)	0.98 ± 0.18	1.01 ± 0.19	1.06 ± 0.15	1.08 ± 0.17
Creatine	3.04 (s)	0.75 ± 0.09	1.63 ± 0.22 *	0.88 ± 0.13 ^#^	0.92 ± 0.16^#^
Leucine + isoleucine	0.95–0.97 (m)	0.37 ± 0.05	0.75 ± 0.03	0.41 ± 0.02	0.45 ± 0.03
Choline	3.20 (s)	0.25 ± 0.02	0.58 ± 0.07 *	0.32 ± 0.08 ^#^	0.37 ± 0.06 ^#^
Betaine	3.89 (s)	1.52 ± 0.13	0.80 ± 0.22 *	1.49 ± 0.16 ^#^	1.42 ± 0.14 ^#^

Multiplicity: s, single; d, double; t, triplet; q, quartet; m, multiplet. * *p* < 0.05, when compared to control; ^#^ *p* < 0.05, when compared to cinnabar.

**Table 3:** ^1^H-NMR-relative integral levels of metabolites in serum samples of groups control, cinnabar, *Coptis chinensis* and berberine.

Metabolites	Chemical Shift (ppm)	Control Group	Cinnabar Group	*Coptis chinensis* Group	Berberine Group
α-Oxoglutarate	2.47 (m)	1.05 ± 0.14	0.52 ± 0.18 *	1.12 ± 0.13 ^#^	1.09 ± 0.11 ^#^
lactate	1.32 (d), 4.14 (q)	4.59 ± 0.83	8.85 ± 1.22 *	5.45 ± 0.95 ^#^	5.96 ± 0.72 ^#^
Trimethylamine-*N*-oxide	3.27 (s)	1.64 ± 0.22	0.98 ± 0.17 *	1.76 ± 0.15 ^#^	1.72 ± 0.23 ^#^
Creatine	3.07 (s)	0.28 ± 0.03	0.65 ± 0.04 *	0.31 ± 0.02 ^#^	0.37 ± 0.04 ^#^
Leucine	0.94 (d)	0.48 ± 0.04	0.82 ± 0.05 *	0.46 ± 0.03 ^#^	0.53 ± 0.04 ^#^
Isoleucine	0.99 (t), 1.02 (d)	0.56 ± 0.06	1.03 ± 0.08 *	0.64 ± 0.05 ^#^	0.67 ± 0.08 ^#^
Alanine	1.50 (d)	1.24 ± 0.09	0.81 ± 0.06 *	1.19 ± 0.08 ^#^	1.13 ± 0.07 ^#^
Pyruvate	2.41 (s)	0.13 ± 0.02	0.28 ± 0.02 *	0.15 ± 0.03 ^#^	0.16 ± 0.04 ^#^
Choline	3.20 (s)	0.24 ± 0.06	0.48 ± 0.03 *	0.18 ± 0.02 ^#^	0.21 ± 0.04 ^#^

Multiplicity: s, single; d, double; t, triplet; q, quartet; m, multiplet. * *p* < 0.05, when compared to control; ^#^ *p* < 0.05, when compared to cinnabar.

## Supplementary Materials

The following are available online. Table S1: The pathway analysis potential target metabolites.
