# Supplementary material for: 1H-NMR-Based Metabonomics of the Protective Effect of Coptis chinensis and Berberine on Cinnabar-Induced Hepatotoxicity and Nephrotoxicity in Rats
Source: Molecules. 2017 Nov 2;22(11):1855. doi: 10.3390/molecules22111855 (PMC6150353; doi:10.3390/molecules22111855)
Supplement: Supplementary file 1 [file molecules-22-01855-s001.pdf]

Table S1. The pathway analysis potential target metabolites

Metabolic pathway	Total	Expected	Hits	Raw p	-Log(p)	Holm adjust	FDR	Impact
Citrate cycle (TCA cycle)	20	0.22825	4	4.93E-05	9.9185	0.00399	0.00399	0.17794
Alanine, aspartate and glutamate metabolism	24	0.27389	4	0.000105	9.1608	0.008406	0.004256	0.00316
Glycine, serine and threonine metabolism	32	0.36519	4	0.000336	7.9973	0.026574	0.009082	0.29197
Pyruvate metabolism	22	0.25107	3	0.001647	6.4086	0.12849	0.033359	0.24337
Glycolysis or Gluconeogenesis	26	0.29672	3	0.002704	5.9129	0.20824	0.043811	0.12753
Methane metabolism	9	0.10271	2	0.004198	5.4732	0.31903	0.056669	0
Valine, leucine and isoleucine biosynthesis	11	0.12553	2	0.006328	5.0628	0.47461	0.073226	0.33333
Glyoxylate and dicarboxylate metabolism	16	0.1826	2	0.013354	4.3159	0.98822	0.13521	0.40741
Butanoate metabolism	20	0.22825	2	0.020589	3.883	1	0.1853	0
Aminoacyl-tRNA biosynthesis	67	0.76462	3	0.037374	3.2868	1	0.30273	0
Cyanoamino acid metabolism	6	0.068474	1	0.066665	2.7081	1	0.4909	0
Taurine and hypotaurine metabolism	8	0.091298	1	0.087945	2.431	1	0.56939	0.42857
Primary bile acid biosynthesis	46	0.52496	2	0.094426	2.3599	1	0.56939	0.05952
Nitrogen metabolism	9	0.10271	1	0.098413	2.3186	1	0.56939	0
Selenoamino acid metabolism	15	0.17118	1	0.1589	1.8395	1	0.80441	0

Pantothenate and CoA biosynthesis	15	0.17118	1	0.1589	1.8395	1	0.80441	0
Propanoate metabolism	20	0.22825	1	0.20637	1.5781	1	0.98329	0
Glutathione metabolism	26	0.29672	1	0.26002	1.347	1	1	0.00573
Porphyrin and chlorophyll metabolism	27	0.30813	1	0.26863	1.3144	1	1	0
Cysteine and methionine metabolism	28	0.31954	1	0.27714	1.2832	1	1	0.02103
Glycerophospholipid metabolism	30	0.34237	1	0.29388	1.2246	1	1	0.02315
Valine, leucine and isoleucine degradation	38	0.43367	1	0.35729	1.0292	1	1	0
Tyrosine metabolism	42	0.47932	1	0.38695	0.94947	1	1	0
Arginine and proline metabolism	44	0.50214	1	0.40129	0.91306	1	1	0
